# Supplementary material for: Exploring Core Microbiota Based on Characteristic Flavor Compounds in Different Fermentation Phases of Sufu
Source: Molecules. 2022 Aug 3;27(15):4933. doi: 10.3390/molecules27154933 (PMC9370341; doi:10.3390/molecules27154933)
Supplement: Supplementary file 1 [file molecules-27-04933-s001.zip › molecules-1835315-supplementary.pdf]

Exploring Core Microbiota Based on Characteristic Flavor Compounds in Different Fermentation Phases of Sufu

Wu Wei[1#], Wang Zhuochen[2#], Xu Boyang[1], Cai Jing[1], Cheng Jianghua[2], Mu Dongdong[1], Wu Xuefeng[1], Li Xingjiang[1]*

[1] School of Food and Biological Engineering, Hefei University of Technology, Hefei, Anhui Province 230009, P.R. China.

[2] Institution of Agricultural Products Processing, Anhui Academy of Agricultural Sciences, Hefei, Anhui Province 230001, China

*Corresponding Author: Li Xingjiang

*Email: lixingjiang1978@hfut.edu.cn

Supplementary Table Guide:

Table S1 Physicochemical parameters values including total acidity, salt content, moisture content and ethanol content in sufu samples, means with different superscript letters are significantly different horizontally (one-way analysis of variance; $p < 0.05$)

Figure S1 Wilcoxon rank-sum test bar plot for distinguishing the differences between two stages of sufu fermentation on genus level, significant value are shown as: * $p < 0.05$，** $p < 0.01$. The red color represent the early stage, and the blue color represent the late stage

Figure S2 Principal component analysis biplot for assessing the variance of sufu during fermentation

Figure S3 Correlation network analysis of characteristic flavor components and dominant genera in the late stage, the red line represents a positive correlation and the blue line represents a negative correlation

Table S1

	Sufu samples						
	d0	d10	d20	d30	d60	d90	d130
Total acidity**(g/100g)**	$0.96±0.05^{f}$	$1.16±0.03^{e}$	$1.26±0.03^{d}$	$1.35±0.05^{c}$	$1.47±0.02^{b}$	$1.55±0.05^{a}$	$1.45±0.01^{b}$
Moisture(%)	$65.37±0.10^{d}$	$66.91±0.07^{b}$	$68.37±0.03^{a}$	$67.03±0.09^{b}$	$66.53±0.48^{bc}$	$66.03±0.64^{c}$	$68.92±0.33^{a}$
Salt(g/100g)	$16.70±0.26^{a}$	$14.37±0.28^{d}$	$15.32±0.35^{c}$	$15.96±0.16^{b}$	$15.35±0.26^{c}$	$15.10±0.34^{c}$	$15.12±0.32^{c}$
Ethanol(mg/g)	$12.67±0.11^{g}$	$18.03±0.27^{c}$	$17.52±0.35^{d}$	$17.27±0.38^{e}$	$16.51±0.16^{f}$	$18.87±0.36^{b}$	$20.24±0.21^{a}$

These values were the mean values of three parallel samples.

Figure S1

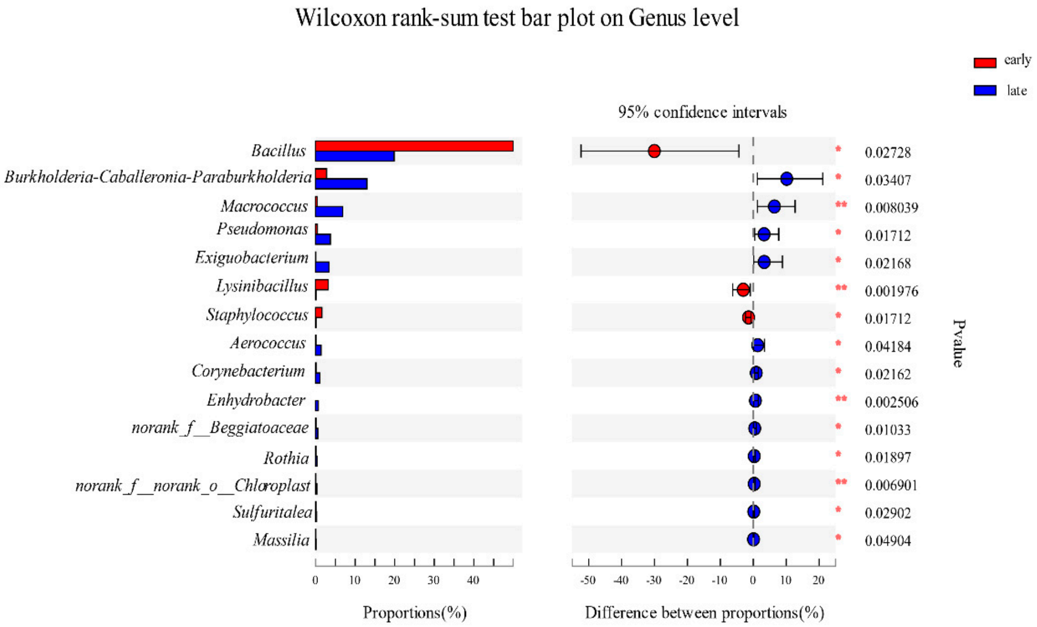

Wilcoxon rank-sum test bar plot on Genus level
early
late
95% confidence intervals
Bacillus
Burkholderia-Caballeronia-Paraburkholderia
Macrococcus
Pseudomonas
Exiguobacterium
Lysinibacillus
Staphylococcus
Aerococcus
Corynebacterium
Enhydrobacter
norank_f__Beggiatoaceae
Rothia
norank_f__norank_o__Chloroplast
Sulfuritalea
Massilia
0.02728
0.03407
0.008039
0.01712
0.02168
0.001976
0.01712
0.04184
0.02162
0.002506
0.01033
0.01897
0.006901
0.02902
0.04904
Pvalue
Proportions(%)
Difference between proportions(%)

Figure S2

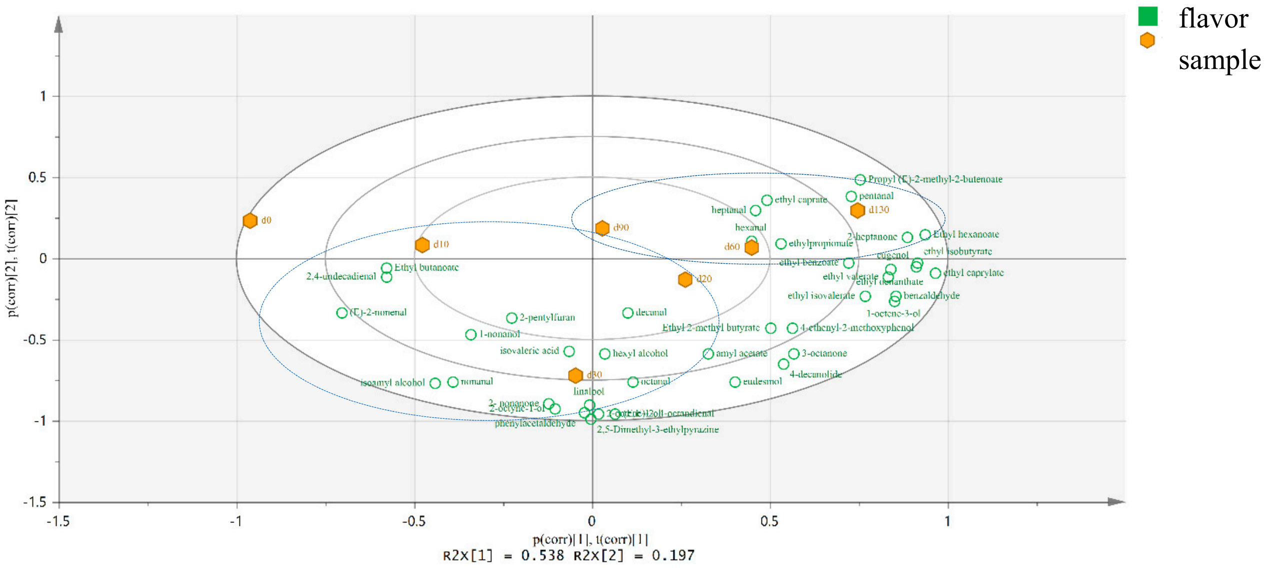
flavor
sample
p(corr)[2], t(corr)[2]
p(corr)[1], t(corr)[1]
R2X[1] = 0.538 R2X[2] = 0.197
1
0.5
0
-0.5
-1
-1.5
-1.5
-1
-0.5
0
0.5
1
d0
d10
d90
d20
d30
d60
d130
Propyl (E)-2-methyl-2-butenoate
pentanal
ethyl caprate
heptanal
hexanal
ethylpropionate
2-heptanone
Ethyl hexanoate
ethyl isobutyrate
eugenol
ethyl benzoate
ethyl caprylate
ethyl valerate
ethyl oenanthate
ethyl isovalerate
benzaldehyde
1-octene-3-ol
Ethyl butanoate
2,4-undecadienal
(E)-2-nonenal
2-pentylfuran
decanal
1-nonanol
Ethyl 2-methyl butyrate
4-ethenyl-2-methoxyphenol
isovaleric acid
hexyl alcohol
amyl acetate
3-octanone
4-decanolide
isoamyl alcohol
nonanal
octanal
eudesmol
linalool
2- nonanone
2-octyne-1-ol
phenylacetaldehyde
2,5-Dimethyl-3-ethylpyrazine

Figure S3

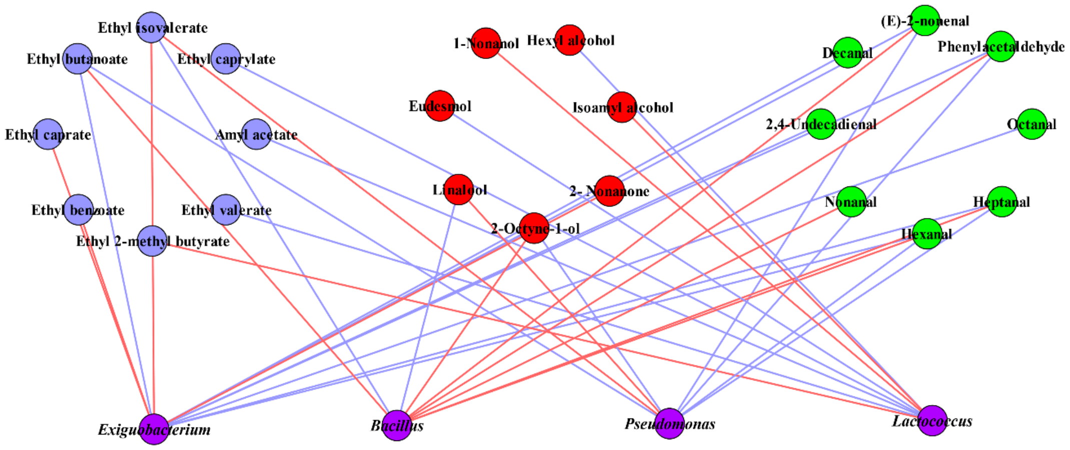

Ethyl isovalerate
Ethyl butanoate
Ethyl caprylate
Ethyl caprate
Amyl acetate
Ethyl benzoate
Ethyl valerate
Ethyl 2-methyl butyrate
1-Nonanol
Hexyl alcohol
Eudesmol
Isoamyl alcohol
Linalool
2- Nonanone
2-Octyne-1-ol
(E)-2-nonenal
Decanal
Phenylacetaldehyde
2,4-Undecadienal
Octanal
Nonanal
Heptanal
Hexanal
Exiguobacterium
Bacillus
Pseudomonas
Lactococcus
